# Recognition of personality disorder and anxiety disorder comorbidity in patients treated for depression in secondary psychiatric care

**DOI:** 10.1371/journal.pone.0227364

**Published:** 2020-01-02

**Authors:** Marie Asp, Daniel Lindqvist, Johan Fernström, Livia Ambrus, Eva Tuninger, Margareta Reis, Åsa Westrin

**Affiliations:** 1 Department of Clinical Sciences Lund, Psychiatry, Lund University, Lund, Sweden; 2 Psychiatric Clinic, Lund, Division of Psychiatry, Lund, Sweden; 3 Department of Clinical Pharmacology, Linköping University, Linköping, Sweden; Assistance Publique Hopitaux de Marseille, FRANCE

## Abstract

**Objectives:**

Depression is a common illness with substantial economic consequences for society and a great burden for affected individuals. About 30% of patients with depression do not respond to repeated treatments. Psychiatric comorbidity is known to affect duration, recurrence and treatment outcome of depression. However, there is a lack of knowledge on the extent to which psychiatric comorbidity is identified in the clinical setting for depressed patients in secondary psychiatric care. Therefore, the aim of this study was to compare the agreement between traditional diagnostic assessment (TDA) and a structured and comprehensive diagnostic procedure (SCDP) for identification of personality and anxiety disorder comorbidity in depressed patients in secondary psychiatric care.

**Methods:**

274 patients aged 18–77 were referred from four secondary psychiatric care clinics in Sweden during 2012–2017. ICD-10 diagnoses according to TDA (mostly unstructured by psychiatric specialist and residents in psychiatry), were retrieved from medical records and compared to diagnoses resulting from the SCDP in the study. This included the Mini International Neuropsychiatric Interview, the Structured Interview for DSM Axis II Personality Disorders and semi-structured questions on psychosocial circumstances, life-events, psychiatric symptoms, psychiatric treatments, substance use, and suicidal and self-harm behaviour. The assessment was carried out by psychiatric specialists or by residents in psychiatry with at least three years of psychiatric training.

**Results:**

SCDP identified personality disorder comorbidity in 43% of the patients compared to 11% in TDA (p<0,0001). Anxiety disorder comorbidity was identified in 58% with SCDP compared to 12% with TDA (p<0,0001).

**Conclusions:**

Important psychiatric comorbidity seems to be unrecognized in depressive patients when using TDA, which is routine in secondary psychiatric care. Comorbidities are better identified using the proposed model involving structured and semi-structured interviews together with clinical evaluations by clinical experts.

## Introduction

Depression is a common illness, affecting nearly 300 million people around the world [1], and is often associated with severe suffering and significant dysfunctions in important areas of life. Moreover, the economic burden of depression is very high, with an estimated global cost of US$ 1 trillion per year due to lost productivity [2, 3]. Treatment resistance is common in depression and is associated with an even more malignant disease course with psychological impairments, poorer occupational outcomes and a higher suicide risk [4].

Less than one third of depressed patients respond to the first line of treatment, and subsequent treatment attempts result in approximately one third of patients not achieving remission [5, 6]. In Sweden, the majority of patients with depression are seen in primary care by general practitioners [7]. Secondary care is provided through regional hospitals by psychiatric specialists. Depressed patients who do not achieve remission in primary care, are often referred by their general practitioner to secondary psychiatric care.

Identifying common comorbidities of depressive disorders in psychiatric patients is considered clinically important due to the association with treatment outcome, duration and recurrence of comorbid disorders and suicide risk. The presence of a comorbid personality disorder has been shown to predict a worse response to antidepressant treatment, persistence, slower remission of depressive disorders and more problem with nonadherence to medication [8–11]. Further, anxiety disorder and substance use disorder comorbidities in clinically depressed patients have repeatedly been reported to affect treatment outcomes [12–15]. Comorbidities with personality disorders and substance use disorders are also associated with an increased suicide risk [16]. Not only personality disorders, but also the individual patterns of affective temperament traits have been shown to be predictors of psychopathology and have been associated with hopelessness and thereby suicide risk [17]. Patients with depression combined with personality disorders and anxiety disorders have also shown more impairment on psychosocial functioning and work impairment than patients with depression only [18–21]. However, comorbid psychiatric disorders may be missed in a clinical setting in both primary and secondary psychiatric care, and personality disorders and anxiety disorders are among those comorbidities that might be neglected [22–24].

Neglected comorbidity and diagnostic inaccuracy could be due to the diagnostic process and procedure. The use of standardized diagnostic interviews has shown significantly better diagnostic accuracy in comparison with traditional diagnostic assessments (TDA), which are often unstructured [25, 26]. For example, personality disorders are often unrecognized when using TDA and are more frequently identified with semi-structured interviews [27]. Furthermore, overall agreement between TDA and standardized diagnostic interviews has been shown to be low to moderate [28, 29]. However, even though structured or semi-structured interviews are highly accepted by both interviewers and patients, such interviews seem to be randomly used in the clinical setting [29]. This might pose a serious clinical problem, as clinically meaningful personality disorders are left unidentified.

Diagnoses according to the International Classification of Diseases 10^th^ revision (ICD-10) registered in the medical record are of great importance for treatment decisions when a patient is treated by different caregivers, particularly when the patient alternates between emergency care, inpatient care and outpatient care. Underestimating personality disorder and anxiety disorder comorbidity in patients treated for depression in secondary psychiatric care may be an issue of patient safety.

There are previous studies comparing TDA with different structured approaches in outpatients and inpatients, mostly on mixed patient material though some on more specific diagnostic groups, such as substance use disorders [23–25, 30–35]. There is, however, no previous study comparing different diagnostic approaches for depressed patients in secondary psychiatric care regarding comorbidity with personality and anxiety disorders.

Since there is a lack of knowledge concerning the ability of different diagnostic approaches to identify comorbidity of depressed patients, the aim of this study was to compare the agreement between TDA reported in secondary psychiatric care medical records to a structured and comprehensive diagnostic procedure (SCDP), with a focus on the recognition of personality disorder and anxiety disorder comorbidity in patients treated for depression.

We hypothesized that personality disorders and anxiety disorders as comorbidities of depression are insufficiently recognized in TDA compared to SCDP in secondary psychiatric care.

## Materials and methods

### Recruitment procedure

This study is part of a larger research project named Genes, Depression and Suicidality (GEND-S). The primary aim of that study is to assess if the frequency of poor, extensive and ultra-rapid metabolizers of *CYP2D6* drug substrates differ between patients who have made suicide attempts and those who have not. Patients who were previously diagnosed with an affective disorder and had an insufficient treatment response were referred to the GEND-S study. In this study insufficient treatment response was defined as not having achieved remission with the previous and ongoing treatments during the current depressive episode. Remission was defined in accordance with Rush et al. as referring only to the nine criterion symptom domains identified in DSM-IV-TR to diagnose a major depressive disorder [36]. The study did not utilize the concept of treatment-resistant depression in inclusion criteria since this concept implies that causes of pseudo-resistance have been ruled out [37]. This was not the case since we suspected that comorbidities may not had been recognized properly.

Patients were referred to the project from four psychiatric clinics in southern Sweden. Patients with clinical unipolar or bipolar depression or suspected clinical depression according to a referring specialist or resident in psychiatry were included. Exclusion criteria were a body mass index less than 15, pregnancy or current liver disease. This study is based on 274 patients referred to the GEND-S project from 51 different specialists or residents in psychiatry between 2012 and 2017. Before inclusion, oral and written information about the purpose of the study was provided and each participant gave their written informed consent. The principles of the Declaration of Helsinki were followed, and the study was approved by the Regional Ethical Review Board in Lund, Sweden. Approval number: 2011/673.

### Traditional diagnostic assessments (TDA)

TDA was defined as the current ICD-10 diagnoses reported in the medical records when the patients entered the study. The routine assessment for these diagnoses is a clinical unstructured interview, though occasionally the Mini International Neuropsychiatric Interview (MINI) [38] or Structured Clinical Interview for DSM-IV Disorders (SCID I) [39] may have been used as a complement to the assessment during the patient’s treatment history. No other structured or semi-structured interviews were used, since MINI and SCID I were the only diagnostic interviews for Axis I disorders that have been translated to Swedish and used in routine care during the period from which medical records were retrieved (2006–2017). The majority of patients previously diagnosed with personality disorders had been assessed using the Structured Clinical Interview for DSM-IV Personality Disorders (SCID-II) [40]. TDA was done by either a psychiatric specialist or a resident in psychiatry.

### Structured and comprehensive diagnostic procedure by clinical experts (SCDP)

After inclusion in the study, the patients were diagnosed according to the Diagnostic and statistical manual of mental disorders 4^th^ edition (DSM-IV-TR) by either a psychiatric specialist or a resident in psychiatry with at least three years of psychiatric training as well as supervision from a senior colleague. Supervision involved the discussion of all patients’ diagnostics. All participating clinicians had undertaken training in the research protocol and the discussion on inter-rater agreement for the instruments used in the study, prior to assessing patients on their own. During the study meetings and clinical discussions were held in order to assure adherence to the research protocol and agreement on the diagnostic procedure.

The diagnostic procedure comprised a standardized research protocol including MINI 6.0 and the Structured Clinical Interview for DSM-IV Personality Disorders (SCID-II). Psychiatric symptoms were assessed using the Comprehensive Psychopathological Rating Scale (CPRS) [41]. All patients completed the self-rating version of the Suicide Assessment Scale (SUAS-S) [42], the self-rating version of the Montgomery-Åsberg Depression Rating Scale (MADRS-S) [43], the UKU side effect scale (UKU-SERS) [44] and the Alcohol Use Disorders Identification Test (AUDIT) [45]. The semi-structured research protocol included questions on previous and current psychosocial circumstances, previous and current psychiatric treatments (psychological, pharmacological and electroconvulsive therapy), on-going and previous psychiatric symptoms, childhood circumstances, traumatic life-events, on-going and previous suicidal and self-harm behavior, on-going and previous alcohol and drug use, and on-going somatic diagnoses and treatments. Patients were also asked about nonadherence and side-effects of earlier and ongoing medication.

### Power calculation and sample size

The prevalence of personality disorders in depressed patients in secondary psychiatric care was expected to be at least 40% based on a previous reasonably comparable study [46]. The expected frequency of personality disorders from medical records was hard to find from previous studies. Frequency in the general population has been calculated to around 12% [47]. Previous studies on frequency of personality disorders in clinical epidemiological studies have been low [48], in fact almost the same as for the general population. However, we expected the frequency to be higher than in epidemiological studies even with TDA. We assumed that many patients would have had a long psychiatric contact and therefore had been, at least to some extent more thoroughly assessed. We therefore did an estimate that 25% of the patients would have been diagnosed with a personality disorder with TDA. For a desired statistical power of 90%, an accepted type 1 error of 5% and a paired one-sided McNemar’s test, this would require a sample of 244 patients.

### Statistical analyses

All statistical analyses were conducted using SPSS statistical software version 24.0 (IBM SPSS Statistics for Macintosh). The significance level was set to p < 0,05. McNemar’s test for paired nominal data was used to compare diagnoses through TDA before the study and diagnoses through SCDP after participation in the study. Pearson’s chi-squared test was used to compare proportions. Variables presented in Table 1 have been assessed to be normally distributed.

**Table 1:** Patient characteristics in the study.

Total number of patients included	274
**Men/women**	93/181
**Suicide attempt (yes/no)**	90/184
**Age (mean, SD)**	38± 14
**Age at first mental health contact (mean, SD)**	25±12
**Number of years in mental health care (mean, SD)**	14±11
**Number of years since first reported depressive episode (mean, SD)**	16±11
**Number of antidepressants prescribed in the patients’ treatment history (mean, SD)**	4 ±2
**Electroconvulsive therapy in treatment history (yes/no)**	46/228
**Psychoterapy in treatment history (yes/no)**	223/49
**Total lines of treatments in treatment history^a^ (mean, SD)**	7±4
**Total MADRS-S score (mean, SD)**	25± 9

^a^ Total lines of treatments include antidepressants, antipsychotics, mood stabilizers, electroconvulsive therapy and psychotherapy.

## Results

Patient characteristics are presented in Table 1.

The mean number of antidepressants tried was four and the mean time between the patient’s first health care contact due to psychiatric symptoms and inclusion to the study was 14 years. The mean total score for MADRS-S was 25 at the day of the research assessment. The different mood disorder diagnoses according to the TDA noted in the medical records versus the diagnostic procedure in the SCDP are given in Table 2.

**Table 2:** Affective disorders for traditional diagnostic assessment (TDA) according to medical records and structured and comprehensive diagnostic procedure (SCDP) according to the study protocol.

Diagnostic group ^a^	Number of patients with the diagnosis according to TDA n = 274	Number of patients with the diagnosis according to SCDP n = 274
**Current mood disorder ^b^**	230	221
*Depression single episode*	31	2
*Depression recurrent*	108	132
*Chronic depression*	6	65
*Depression not otherwise specified*	31	5
*Dysthymia*	22	63
*Bipolar disorder*, *depressive episode*	42	28
**Current mixed anxiety and depression^c^**	30	-
**Mood disorders, currently in remission**	-	50
*Depression*, *recurrent*, *currently in remission*	-	34
*Bipolar disorder*, *currently in remission*	2	16
**No affective disorder**	14	3

^a^ Patients could be assigned more than one diagnosis.

^b^ For bipolar disorder, only a depressive episode is included.

^c^ Mixed anxiety and depression is not considered a mood disorder in this study.

According to the medical records, 230 of 274 patients were diagnosed with current clinical depression in TDA, compared to 221 of 274 patients in the SCDP.

Personality disorders, anxiety disorders, ADHD, eating disorders and substance use disorders were all significantly more common (p < 0,0001) in the SCDP than in the TDA (Table 3). There was also a statistically significant difference in the overall number of patients with no comorbidity according to TDA comparted to SCDP (p<0,001).

**Table 3:** Psychiatric comorbidity compared for traditional diagnostic assessment (TDA) according to medical records and structured and comprehensive diagnostic procedure (SCDP) according to the study protocol.

	*Mood disorder diagnosis according to TDA and SCDP ^a^*	*Anxiety Disorders*	*Eating* *Disorders*	*Autism*	*ADHD*	*Substance use* *disorders*	*Personality disorders*	*No comorbidity*
**Total number of comorbid diagnoses**	**TDA** **n = 274**	**33**	**3**	**1**	**14**	**4**	**30**	**197**
	**SCDP** **n = 274**	159***^b^	22 ***^b^	**4**^c^	26***^b^	16***^b^	119***^b^	62***^d^
*Recurrent depression*	TDA n = 108	16	2	1	7	1	7	77
	SCDP n = 166	102	17	2^c^	12	10	73	27
*Chronic depression*	TDA n = 6	1	-	-	-	-	-	5
	SCDP n = 65	46	3	-	2	4	35	10
*Dysthymic disorder*	TDA n = 22	3	-	-	1	-	2	16
	SCDP n = 63	45	3	3^c^	3	4	31	9
*Bipolar disorder*	TDA n = 44	3	-	-	2	2	10	30
	SCDP n = 44	26	2	1^c^	5	2	18	11

^a^ Both patients with current depression and those in current remission are included in the table.

^b^ Statistical analysis has compared comorbidity for “total” in TDA versus SCDP for each diagnosis except autism. *p* values were calculated using McNemar’s test. *** = p<0,0001.

^c^ One case of diagnosed autism and the other cases from the SCDP were highly suspected. Interview with relatives was considered necessary to confirm the diagnosis and this was not done as part of the study procedure.

^d^ Statistical analysis has compared “no comorbidity” for TDA and SCDP. *p* value was calculated using Chi-square test. *p*<0,001.

Details of personality disorder diagnoses for both TDA and SCDP are given in Table 4.

**Table 4:** Number of patients with personality disorders for traditional diagnostic assessment (TDA) according to medical records and structured and comprehensive diagnostic procedure (SCDP) according to the study protocol.

Personality disorder diagnosis^a^	TDA n = 274	SCDP n = 274
**All patients**	**30**	119***^b^
Cluster A	-	5
**Cluster B**	**18**	45***^b^
**Cluster C**	**3**	47***^b^
Cluster A mixed	-	1
Cluster B mixed	-	14
Cluster C mixed	-	12
Mixed all clusters	-	1
Mixed cluster A+B	-	1
Mixed cluster A+C	-	1
Mixed cluster B+C	-	5
Paranoid	-	2
Schizoid	-	1
Schizotypal	-	-
Borderline	18	19
Antisocial	-	1
Narcissistic	-	4
Histrionic	-	1
Avoidant	2	24
Dependent	1	-
Obsessive compulsive	-	4
Not otherwise specified	9	27

^a^Both patients with current depression and those in current remission are included.

^b^Statistical analysis has been performed comparing TDA and SCDP for “All patients”, Cluster B” and “Cluster C”. *p* values were calculated using McNemar’s test. *** = p<0,0001.

Particularly, a cluster B or cluster C personality disorder was more commonly missed in the TDA compared to the SCDP (p < 0,0001). Avoidant personality disorder and borderline personality disorder were the two most common isolated personality disorders in the SCDP. Multiple/mixed personality disorders occurred in 29% of the patients diagnosed with personality disorders in the SCDP. Among the patients diagnosed with mixed cluster B personality disorders, all patients met the criteria for borderline personality disorder plus one other cluster B personality disorder. Among the patients with mixed cluster C personality disorders all patients met the criteria for avoidant personality disorder as well as one other cluster C personality disorder. Panic disorder, social phobia and generalized anxiety disorder were the most common anxiety disorders given in the SCDP (Table 5).

**Table 5:** Number of patients with different anxiety disorders for traditional diagnostic assessment (TDA) according to medical records and structured and comprehensive diagnostic procedure (SCDP) according to the study protocol.

Diagnosis^a^	TDA n = 274	SCDP n = 274
**All anxiety disorders**	**33**	159***^b^
Social phobia	4	71
Panic disorder	1	47
Generalized anxiety disorder	21	69
Obsessive compulsive disorder	5	30
Posttraumatic stress disorder	3	16

^a^Both patients with current depression and those in current remission are included.

^b^Statistical analysis has compared “All anxiety disorders” in TDA versus SCDP.

*p* values were calculated using McNemar’s test. *** = p<0,0001.

## Discussion

This study showed that there is a lack of personality and anxiety disorder comorbidity assessments in patients treated for depression in current Swedish secondary psychiatric care. This shortcoming demands attention, as earlier studies have shown that without a thorough assessment, psychiatric comorbidity may go unrecognized [24] and that personality disorders as well as anxiety disorder comorbidities affect the treatment outcome of depression [8, 10, 13, 14, 49–51].

The overall findings of this study are in line with earlier studies showing that TDA is more unreliable, as it often fails to identify diagnostic criteria for personality disorders [25]. The frequency of personality disorders identified with TDA in this study was surprisingly low and in the same range as found for the general population, despite long psychiatric contacts for the included patients. A difference between this study and previous work is that this study focused on patients with depression and insufficient treatment response. To our knowledge, earlier studies have not examined this specific group when comparing TDA with a more structured diagnostic approach for the assessment of psychiatric comorbidity. One previous report compared clinical and research assessment for diagnosis and suicide risk assessment for depressed patients but without a focus on comorbidity [52].

The rate of personality disorders in the SCDP were in the lower range though in accordance with previous reports. A literature review by Beckwith et al. showed prevalence estimates of personality disorders in psychiatric outpatients of 45–51% in the USA and 40–92% in Europe [53]. Most reports, however, were in the range of 40–60%. In a report from a European multi-center study on treatment resistant depression by Soury et al., personality disorders were seen in 37% of patients without treatment resistance and in 51% with treatment resistance [54]. The wide range of prevalence rates in previous reports is most likely due to methodological differences in assessment methods and population samples. Self-rating questionnaire-based personality disorder assessments show consistently higher estimates than interview-based assessments [55]. In this study, the SCID II semi-structured interview was used for the assessment of personality disorders. The SCID II has repeatedly been shown to have good inter-rater and internal consistency reliability [56, 57]. When comparing standardized instruments to assess personality pathology in depressed patients it has been concluded that semi-structured interviews seem to have more validity than self-rated questionnaires [58].

The rate of anxiety disorders identified with SCDP was in accordance with previous studies in patients with depression. Howland et al. showed a prevalence of 64% for anxiety disorders as comorbidities of depression in specialty care. Data from their study were obtained as part of the Sequenced Treatment Alternatives to Relieve Depression (STAR*D) trial [13].

Only 23% of the patients assessed with SCDP were without psychiatric comorbidity compared to 72% for TDA. Earlier studies have not presented figures to compare with for total comorbidity for this group of patients. However, almost all research studies use structured or semi-structured interviews to assess diagnostic criteria and such interviews have sometimes been argued to identify too many diagnoses that may not be relevant. The agreement between the TDA and diagnoses obtained using the MINI has been investigated and the result show moderate agreement, though the MINI identified more diagnoses per patient than the TDA [29]. That the MINI identified a higher number of diagnoses per patient could be due to the fact that comorbidity is more easily identified, though this could also be related to an overlap of disorder criteria due to shortcomings in the diagnostic system. It is worth noticing that structured interviews in the research are often not undertaken by a clinical psychiatrist, but by lay interviewers [29, 59]. The experienced clinician may be better at identifying the diagnoses most relevant to the patient, which would result in a smaller number of diagnoses per patient when experienced clinicians use the MINI. In this study, we have only assigned multiple diagnoses when there has been an evident clinical comorbidity. From the results, it can be assumed that for most patients, depression alone does not explain the treatment difficulties and that comorbidities could play an important role.

SCDP identified less patients with current depression (221) compared to TDA (230), although the difference is small and not statistically significant. We could not rule out that some patients assessed with SCDP have residual symptoms of depression but did not reach the diagnostic threshold. Another potential reason for less patients being diagnosed with current depression in the SCDP group could be that the detected comorbidity may better explain some symptoms that have earlier been interpreted as primarily depressive. In this study we focused on fulfilment of diagnostic criteria for depression and comorbidities only and did not register residual symptoms of depression or depression in partial remission.

Regarding the concept of insufficient treatment response defined as patients not having achieved remission on earlier and ongoing treatments for the current depressive episode, there might have been insufficient number of treatment attempts for some patients. Especially for patients who have not received psychotherapy, the treatment difficulties might be related to insufficient treatment itself. This could be a cause of pseudo-resistance, which includes identification of treatment attempts, doses and treatment duration as well as identification of psychiatric and somatic comorbidities and non-compliance [37]. This illustrates the importance of adequately designed stepped-care strategies for depression, improving outcome and having a highly beneficial cost-effectiveness compared to usual care guided by clinicians’ choices [60–62]. Thus, in this study, one important reason for patients not achieving remission could be both insufficient treatment attempts and earlier unrecognized psychiatric comorbidity. Another reason could be non-compliance. A more clear-cut study definition on lines of treatment, doses or mechanisms of action for pharmacological treatment could have facilitated comparison with other studies on treatment resistant depression.

One potential limitation of the study is that it was not primarily designed to examine the agreement between TDA noted in the medical records and SCDP by trained clinical experts. It is thus important to point out that the analyses carried out in this study are exploratory and should be interpreted with care. However, the SCDP used in the study confirmed that many of the patients suffered from depressive disorders and revealed long treatment histories with several lines of treatments. Furthermore, many of the patients were referred to the project because the psychiatrist in charge of their treatment regarded their depressive disorder as difficult to treat. Accordingly, the patients in the study represent a group who would probably benefit from a thorough clinical assessment earlier in their treatment history, as the personality or anxiety disorder comorbidities might have been important clinical underlying problems which affect the prognosis of the depressive disorder.

Another potential limitation of the study is that the ICD system was used to assign diagnoses in the medical records while the DSM system was used in the SCDP. Although the two systems are often comparable, as in the case of personality disorders where there is strong agreement between the criteria according to both ICD-10 and DSM-IV [63], there can be inconsistencies. Regarding anxiety and depressive disorders, for example, there is one important difference between the two diagnostic systems. The ICD includes a diagnosis of “mixed anxiety and depressive disorder” (F41.2), which the DSM does not. This diagnosis has been criticized due to problems with the test-retest situation, where most patients with the mixed disorder were reclassified into depression [64].

Other potentially important limitations to the study include the fact that no drug screening or blood tests were performed in order to check for alcohol or drug abuse. Diagnoses of substance abuse are lower in the study population compared to known estimates of both the general population [65, 66] and of patients with depression in both primary and secondary psychiatric care [15, 67, 68]. One obvious explanation for this is under-reporting, although alcohol and drug consumption rate was carefully asked for. Another possibility is that patients with depression and known substance use disorders were not referred to the study or may not have been interested in taking part. However, even if the rate of substance abuse was lower than expected in the study, significantly more patients were identified as having a substance use disorder simply by asking detailed and structured questions about it, highlighting the need for such questions to be routinely used in mental health care. This is especially important since substance use disorder require specific treatment in order to improve both adherence and treatment outcome for depression [65]. It is also important to identify and treat substance use disorders in order to lower suicide risk [16].

Furthermore, no cognitive capability tests were conducted, possibly missing people with intellectual disabilities or abilities in the lower normal range. Such individuals could more frequently be affected by depressive symptoms and might be at higher risk of experiencing difficulties in interpersonal functioning. For some patients, assessment of intellectual functions was recommended after participation, as cognitive difficulties were observed during the interview. Additionally, no relatives were interviewed in the study. Overall, interviewers were careful to assign a diagnosis of personality disorder when there was a lack of information regarding functioning earlier in life. Such information can sometimes be hard to verify without interviewing relatives. Thus, the prevalence of personality disorders in the study would probably have slightly increased if relatives had been interviewed.

Despite the limitations described above, this study shows that psychiatric comorbidities, especially personality disorders and anxiety disorders, but also substance use disorders, are often unrecognized in patients with depression and poor treatment outcome in secondary psychiatric care. This could have serious clinical implications where the lack of identification of psychiatric comorbidities could result in the substantial delay of correct treatment. Such delay might also have a negative impact on the prognosis of the depressive episode. Many of the patients included in our study have had their psychiatric symptoms for many years and one reason for the lack of improvement could be the presence of untreated comorbidity. It is of special importance to note that our results suggest that cluster B and cluster C personality disorders may go unrecognized in routine psychiatric care. This is of great clinical importance as patients with neglected borderline personality disorders may not receive proper treatment, such as dialectic behavioral therapy, which has repeatedly been shown to be effective [69]. Individuals suffering from personality disorders within cluster C may also benefit from a treatment plan that addresses difficulties in interpersonal functioning. A thorough diagnostic assessment can also help the patient to better understand their difficulties and to shift the focus to treatment options other than pharmacological treatment.

In conclusion it can be argued that there is a need for a more structured diagnostic approach to patients with depression and poor treatment outcome in secondary psychiatric care. A structured approach can more easily identify important psychiatric comorbidity including personality disorders. This study also suggests that such structured diagnostic procedures might reduce the frequency of unspecified diagnoses. To improve the accuracy of the diagnostic procedure for depressive disorders, we suggest that clinical experts use structured and semi-structured interviews as part of the clinical assessment. Clinical experts have appropriate training to interpret the results of the standardized diagnostic interviews in a clinically relevant context. It is also important to have extensive information on the course of the psychiatric symptoms, earlier life events, and social factors since such information can help clarify diagnostic difficulties. Whether or not the general criteria for personality disorders are fulfilled requires extensive information about the course of the psychiatric symptoms and level of functioning. Earlier life events could also influence the depressive disorder. Interesting research has presented a possible link between childhood trauma and abnormal brain connectivity in major depressive disorder [70]. This requires further study but addresses one aspect of the problem with the heterogeneity of depression.

Further research including prospective studies is needed to establish if the correct identification and early treatment of comorbidities influence treatment outcome and prognosis in depressive disorders. Specifically, it would be of interest to prospectively assess treatment outcome and disease course in patients evaluated with TDA versus SCDP, as a means of determining the clinical impact of structured diagnostic procedures on long-term disease trajectories. Because of the high comorbidity frequency in depression, future studies testing novel antidepressant treatments, both pharmacological and psychotherapeutical, should take comorbidity into account. This is important in order to improve the prognosis of the depressive disorder and also address important risk factors for suicide, to improve quality of life and the cost effectiveness of the mental health care system.

## References

[1] Kessler RC, Aguilar-Gaxiola S, Alonso J, Chatterji S, Lee S, Ormel J, et al The global burden of mental disorders: an update from the WHO World Mental Health (WMH) surveys. Epidemiologia e psichiatria sociale. 2009;18(1):23–33. 10.1017/s1121189x00001421 19378696 PMC3039289

[2] Greenberg PE, Fournier AA, Sisitsky T, Pike CT, Kessler RC. The economic burden of adults with major depressive disorder in the United States (2005 and 2010). The Journal of clinical psychiatry. 2015;76(2):155–62. 10.4088/JCP.14m09298 25742202

[3] Chisholm D, Sweeny K, Sheehan P, Rasmussen B, Smit F, Cuijpers P, et al Scaling-up treatment of depression and anxiety: a global return on investment analysis. The lancet Psychiatry. 2016;3(5):415–24. 10.1016/S2215-0366(16)30024-4 27083119

[4] Holtzheimer PE, Mayberg HS. Stuck in a rut: rethinking depression and its treatment. Trends in neurosciences. 2011;34(1):1–9. 10.1016/j.tins.2010.10.004 21067824 PMC3014414

[5] Souery D, Amsterdam J, de Montigny C, Lecrubier Y, Montgomery S, Lipp O, et al Treatment resistant depression: methodological overview and operational criteria. European neuropsychopharmacology: the journal of the European College of Neuropsychopharmacology. 1999;9(1–2):83–91. 10082232 10.1016/s0924-977x(98)00004-2

[6] Rush AJ, Trivedi MH, Wisniewski SR, Nierenberg AA, Stewart JW, Warden D, et al Acute and longer-term outcomes in depressed outpatients requiring one or several treatment steps: a STAR*D report. The American journal of psychiatry. 2006;163(11):1905–17. 10.1176/ajp.2006.163.11.1905 17074942

[7] Sundquist J, Ohlsson H, Sundquist K, Kendler KS. Common adult psychiatric disorders in Swedish primary care where most mental health patients are treated. BMC Psychiatry. 2017;17(1):235 10.1186/s12888-017-1381-4 28666429 PMC5493066

[8] Skodol AE, Grilo CM, Keyes KM, Geier T, Grant BF, Hasin DS. Relationship of personality disorders to the course of major depressive disorder in a nationally representative sample. The American journal of psychiatry. 2011;168(3):257–64. 10.1176/appi.ajp.2010.10050695 21245088 PMC3202962

[9] Grilo CM, Sanislow CA, Shea MT, Skodol AE, Stout RL, Gunderson JG, et al Two-year prospective naturalistic study of remission from major depressive disorder as a function of personality disorder comorbidity. Journal of consulting and clinical psychology. 2005;73(1):78–85. 10.1037/0022-006X.73.1.78 15709834 PMC3289285

[10] Mulder RT. Personality pathology and treatment outcome in major depression: a review. The American journal of psychiatry. 2002;159(3):359–71. 10.1176/appi.ajp.159.3.359 11869996

[11] Pompili M, Venturini P, Palermo M, Stefani H, Seretti ME, Lamis DA, et al Mood disorders medications: predictors of nonadherence—review of the current literature. Expert review of neurotherapeutics. 2013;13(7):809–25. 10.1586/14737175.2013.811976 23898852

[12] Bagby RM, Ryder AG, Cristi C. Psychosocial and clinical predictors of response to pharmacotherapy for depression. Journal of psychiatry & neuroscience: JPN. 2002;27(4):250–7. 12174734 PMC161659

[13] Howland RH, Rush AJ, Wisniewski SR, Trivedi MH, Warden D, Fava M, et al Concurrent anxiety and substance use disorders among outpatients with major depression: clinical features and effect on treatment outcome. Drug and alcohol dependence. 2009;99(1–3):248–60. 10.1016/j.drugalcdep.2008.08.010 18986774

[14] Campbell-Sills L, Sherbourne CD, Roy-Byrne P, Craske MG, Sullivan G, Bystritsky A, et al Effects of co-occurring depression on treatment for anxiety disorders: analysis of outcomes from a large primary care effectiveness trial. The Journal of clinical psychiatry. 2012;73(12):1509–16. 10.4088/JCP.12m07955 23290323 PMC3692282

[15] Davis LL, Rush JA, Wisniewski SR, Rice K, Cassano P, Jewell ME, et al Substance use disorder comorbidity in major depressive disorder: an exploratory analysis of the Sequenced Treatment Alternatives to Relieve Depression cohort. Comprehensive psychiatry. 2005;46(2):81–9. 10.1016/j.comppsych.2004.07.025 15723023

[16] Hawton K, Casanas ICC, Haw C, Saunders K. Risk factors for suicide in individuals with depression: a systematic review. Journal of affective disorders. 2013;147(1–3):17–28. 10.1016/j.jad.2013.01.004 23411024

[17] Pompili M, Rihmer Z, Akiskal H, Amore M, Gonda X, Innamorati M, et al Temperaments mediate suicide risk and psychopathology among patients with bipolar disorders. Comprehensive psychiatry. 2012;53(3):280–5. 10.1016/j.comppsych.2011.04.004 21641589

[18] Skodol AE, Grilo CM, Pagano ME, Bender DS, Gunderson JG, Shea MT, et al Effects of personality disorders on functioning and well-being in major depressive disorder. J Psychiatr Pract. 2005;11(6):363–8. 10.1097/00131746-200511000-00002 16304504 PMC2548415

[19] Gulec MY, Hocaoglu C. Relationship between personality and disability in patients with major depressive disorder. Isr J Psychiatry Relat Sci. 2011;48(2):123–8. 22120448

[20] Gili M, Garcia Toro M, Armengol S, Garcia-Campayo J, Castro A, Roca M. Functional impairment in patients with major depressive disorder and comorbid anxiety disorder. Can J Psychiatry. 2013;58(12):679–86. 10.1177/070674371305801205 24331287

[21] Hendriks SM, Spijker J, Licht CM, Hardeveld F, de Graaf R, Batelaan NM, et al Long-term work disability and absenteeism in anxiety and depressive disorders. Journal of affective disorders. 2015;178:121–30. 10.1016/j.jad.2015.03.004 25805404

[22] De Carlo V, Calati R, Serretti A. Socio-demographic and clinical predictors of non-response/non-remission in treatment resistant depressed patients: A systematic review. Psychiatry research. 2016;240:421–30. 10.1016/j.psychres.2016.04.034 27155594

[23] Ramirez Basco M, Bostic JQ, Davies D, Rush AJ, Witte B, Hendrickse W, et al Methods to improve diagnostic accuracy in a community mental health setting. The American journal of psychiatry. 2000;157(10):1599–605. 10.1176/appi.ajp.157.10.1599 11007713

[24] Zimmerman M, Mattia JI. Psychiatric diagnosis in clinical practice: is comorbidity being missed? Comprehensive psychiatry. 1999;40(3):182–91. 10.1016/s0010-440x(99)90001-9 10360612

[25] Miller PR, Dasher R, Collins R, Griffiths P, Brown F. Inpatient diagnostic assessments: 1. Accuracy of structured vs. unstructured interviews. Psychiatry research. 2001;105(3):255–64. 10.1016/s0165-1781(01)00317-1 11814544

[26] Miller PR. Inpatient diagnostic assessments: 3. Causes and effects of diagnostic imprecision. Psychiatry research. 2002;111(2–3):191–7. 10.1016/s0165-1781(02)00147-6 12374636

[27] Zimmerman M, Rothschild L, Chelminski I. The prevalence of DSM-IV personality disorders in psychiatric outpatients. The American journal of psychiatry. 2005;162(10):1911–8. 10.1176/appi.ajp.162.10.1911 16199838

[28] Rettew DC, Lynch AD, Achenbach TM, Dumenci L, Ivanova MY. Meta-analyses of agreement between diagnoses made from clinical evaluations and standardized diagnostic interviews. International journal of methods in psychiatric research. 2009;18(3):169–84. 10.1002/mpr.289 19701924 PMC6878243

[29] Verhoeven FEA, Swaab L, Carlier IVE, van Hemert AM, Zitman FG, Ruhe HG, et al Agreement between clinical and MINI diagnoses in outpatients with mood and anxiety disorders. Journal of affective disorders. 2017;221:268–74. 10.1016/j.jad.2017.06.041 28662459

[30] Komiti AA, Jackson HJ, Judd FK, Cockram AM, Kyrios M, Yeatman R, et al A comparison of the Composite International Diagnostic Interview (CIDI-Auto) with clinical assessment in diagnosing mood and anxiety disorders. The Australian and New Zealand journal of psychiatry. 2001;35(2):224–30. 10.1046/j.1440-1614.2001.00868.x 11284905

[31] Kranzler HR, Kadden RM, Burleson JA, Babor TF, Apter A, Rounsaville BJ. Validity of psychiatric diagnoses in patients with substance use disorders: is the interview more important than the interviewer? Comprehensive psychiatry. 1995;36(4):278–88. 10.1016/s0010-440x(95)90073-x 7554872

[32] Shear MK, Greeno C, Kang J, Ludewig D, Frank E, Swartz HA, et al Diagnosis of nonpsychotic patients in community clinics. The American journal of psychiatry. 2000;157(4):581–7. 10.1176/appi.ajp.157.4.581 10739417

[33] Rosenman SJ, Korten AE, Levings CT. Computerised diagnosis in acute psychiatry: validity of CIDI-Auto against routine clinical diagnosis. Journal of psychiatric research. 1997;31(5):581–92. 10.1016/s0022-3956(97)00032-0 9368199

[34] Steiner JL, Tebes JK, Sledge WH, Walker ML. A comparison of the structured clinical interview for DSM-III-R and clinical diagnoses. The Journal of nervous and mental disease. 1995;183(6):365–9. 10.1097/00005053-199506000-00003 7798084

[35] Taggart C, O'Grady J, Stevenson M, Hand E, Mc Clelland R, Kelly C. Accuracy of diagnosis at routine psychiatric assessment in patients presenting to an accident and emergency department. General hospital psychiatry. 2006;28(4):330–5. 10.1016/j.genhosppsych.2006.05.002 16814633

[36] Rush AJ, Kraemer HC, Sackeim HA, Fava M, Trivedi MH, Frank E, et al Report by the ACNP Task Force on response and remission in major depressive disorder. Neuropsychopharmacology: official publication of the American College of Neuropsychopharmacology. 2006;31(9):1841–53. 16794566 10.1038/sj.npp.1301131

[37] Bennabi D, Charpeaud T, Yrondi A, Genty JB, Destouches S, Lancrenon S, et al Clinical guidelines for the management of treatment-resistant depression: French recommendations from experts, the French Association for Biological Psychiatry and Neuropsychopharmacology and the fondation FondaMental. BMC psychiatry. 2019;19(1):262 10.1186/s12888-019-2237-x 31455302 PMC6712810

[38] Sheehan DV, Lecrubier Y, Sheehan KH, Amorim P, Janavs J, Weiller E, et al The Mini-International Neuropsychiatric Interview (M.I.N.I.): the development and validation of a structured diagnostic psychiatric interview for DSM-IV and ICD-10. The Journal of clinical psychiatry. 1998;59 Suppl 20:22–33;quiz 4–57. 9881538

[39] First MB, Spitzer R.L., Gibbon M., & Williams J.B.W.. Structured Clinical Interview for DSM-IV Axis I Disorders (SCID I).: New York: Biometric Research Department; 1997.

[40] First M, Gibbon M, Spitzer RL, Williams JBW, Benjamin LS. Structured Clinical Interview for DSM-IV Axis II Personality Disorders, (SCID-II). Washington, D.C.: American Psychiatric Press, Inc; 1997.

[41] Asberg M, Montgomery SA, Perris C, Schalling D, Sedvall G. A comprehensive psychopathological rating scale. Acta psychiatrica Scandinavica Supplementum. 1978(271):5–27. 10.1111/j.1600-0447.1978.tb02357.x 277059

[42] Nimeus A, Hjalmarsson Stahlfors F, Sunnqvist C, Stanley B, Traskman-Bendz L. Evaluation of a modified interview version and of a self-rating version of the Suicide Assessment Scale. European psychiatry: the journal of the Association of European Psychiatrists. 2006;21(7):471–7. 16545548 10.1016/j.eurpsy.2006.01.011

[43] Svanborg P, Asberg M. A comparison between the Beck Depression Inventory (BDI) and the self-rating version of the Montgomery Asberg Depression Rating Scale (MADRS). Journal of affective disorders. 2001;64(2–3):203–16. 10.1016/s0165-0327(00)00242-1 11313087

[44] Lingjaerde O, Ahlfors UG, Bech P, Dencker SJ, Elgen K. The UKU side effect rating scale. A new comprehensive rating scale for psychotropic drugs and a cross-sectional study of side effects in neuroleptic-treated patients. Acta psychiatrica Scandinavica Supplementum. 1987;334:1–100. 10.1111/j.1600-0447.1987.tb10566.x 2887090

[45] Bergman H, Kallmen H, Rydberg U, Sandahl C. [Ten questions about alcohol as identifier of addiction problems. Psychometric tests at an emergency psychiatric department]. Lakartidningen. 1998;95(43):4731–5. 9821761

[46] Newton-Howes G, Tyrer P, Anagnostakis K, Cooper S, Bowden-Jones O, Weaver T. The prevalence of personality disorder, its comorbidity with mental state disorders, and its clinical significance in community mental health teams. Soc Psychiatry Psychiatr Epidemiol. 2010;45(4):453–60. 10.1007/s00127-009-0084-7 19543844

[47] Volkert J, Gablonski TC, Rabung S. Prevalence of personality disorders in the general adult population in Western countries: systematic review and meta-analysis. The British journal of psychiatry: the journal of mental science. 2018:1–7. 10.1192/bjp.2018.202 30261937

[48] Oldham JM, Skodol AE. Personality disorders in the public sector. Hosp Community Psychiatry. 1991;42(5):481–7. 10.1176/ps.42.5.481 2060913

[49] Angstman KB, Seshadri A, Marcelin A, Gonzalez CA, Garrison GM, Allen JS. Personality Disorders in Primary Care: Impact on Depression Outcomes Within Collaborative Care. Journal of primary care & community health. 2017;8(4):233–8. 10.1177/2150131917714929 PMC5932731 28613090

[50] Marchesi C, De Panfilis C, Cantoni A, Fonto S, Giannelli MR, Maggini C. Personality disorders and response to medication treatment in panic disorder: a 1-year naturalistic study. Progress in neuro-psychopharmacology & biological psychiatry. 2006;30(7):1240–5. 16678956 10.1016/j.pnpbp.2006.03.010

[51] Grilo CM, Stout RL, Markowitz JC, Sanislow CA, Ansell EB, Skodol AE, et al Personality disorders predict relapse after remission from an episode of major depressive disorder: a 6-year prospective study. The Journal of clinical psychiatry. 2010;71(12):1629–35. 10.4088/JCP.08m04200gre 20584514 PMC4615714

[52] Bongiovi-Garcia ME, Merville J, Almeida MG, Burke A, Ellis S, Stanley BH, et al Comparison of clinical and research assessments of diagnosis, suicide attempt history and suicidal ideation in major depression. Journal of affective disorders. 2009;115(1–2):183–8. 10.1016/j.jad.2008.07.026 18814917 PMC3785082

[53] Beckwith H, Moran PF, Reilly J. Personality disorder prevalence in psychiatric outpatients: a systematic literature review. Personality and mental health. 2014;8(2):91–101. 10.1002/pmh.1252 24431304

[54] Souery D, Oswald P, Massat I, Bailer U, Bollen J, Demyttenaere K, et al Clinical factors associated with treatment resistance in major depressive disorder: results from a European multicenter study. The Journal of clinical psychiatry. 2007;68(7):1062–70. 10.4088/jcp.v68n0713 17685743

[55] Friborg O, Martinsen EW, Martinussen M, Kaiser S, Overgard KT, Rosenvinge JH. Comorbidity of personality disorders in mood disorders: a meta-analytic review of 122 studies from 1988 to 2010. Journal of affective disorders. 2014;152–154:1–11. 10.1016/j.jad.2013.08.023 24120406

[56] Maffei C, Fossati A, Agostoni I, Barraco A, Bagnato M, Deborah D, et al Interrater reliability and internal consistency of the structured clinical interview for DSM-IV axis II personality disorders (SCID-II), version 2.0. Journal of personality disorders. 1997;11(3):279–84. 10.1521/pedi.1997.11.3.279 9348491

[57] Lobbestael J, Leurgans M, Arntz A. Inter-rater reliability of the Structured Clinical Interview for DSM-IV Axis I Disorders (SCID I) and Axis II Disorders (SCID II). Clinical psychology & psychotherapy. 2011;18(1):75–9. 20309842 10.1002/cpp.693

[58] Alnaes R, Torgersen S. DSM-III symptom disorders (Axis I) and personality disorders (Axis II) in an outpatient population. Acta psychiatrica Scandinavica. 1988;78(3):348–55. 10.1111/j.1600-0447.1988.tb06346.x 3195356

[59] Helzer JE, Robins LN, McEvoy LT, Spitznagel EL, Stoltzman RK, Farmer A, et al A comparison of clinical and diagnostic interview schedule diagnoses. Physician reexamination of lay-interviewed cases in the general population. Archives of general psychiatry. 1985;42(7):657–66. 10.1001/archpsyc.1985.01790300019003 4015307

[60] Meeuwissen JAC, Feenstra TL, Smit F, Blankers M, Spijker J, Bockting CLH, et al The cost-utility of stepped-care algorithms according to depression guideline recommendations—Results of a state-transition model analysis. Journal of affective disorders. 2019;242:244–54. 10.1016/j.jad.2018.08.024 30216769

[61] Firth N, Barkham M, Kellett S. The clinical effectiveness of stepped care systems for depression in working age adults: a systematic review. Journal of affective disorders. 2015;170:119–30. 10.1016/j.jad.2014.08.030 25240141

[62] van Straten A, Hill J, Richards DA, Cuijpers P. Stepped care treatment delivery for depression: a systematic review and meta-analysis. Psychological medicine. 2015;45(2):231–46. 10.1017/S0033291714000701 25065653

[63] Ottosson H, Bodlund O, Ekselius L, Grann M, von Knorring L, Kullgren G, et al DSM-IV and ICD-10 personality disorders: a comparison of a self-report questionnaire (DIP-Q) with a structured interview. European psychiatry: the journal of the Association of European Psychiatrists. 1998;13(5):246–53. 19698634 10.1016/S0924-9338(98)80013-8

[64] Moller HJ, Bandelow B, Volz HP, Barnikol UB, Seifritz E, Kasper S. The relevance of 'mixed anxiety and depression' as a diagnostic category in clinical practice. European archives of psychiatry and clinical neuroscience. 2016;266(8):725–36. 10.1007/s00406-016-0684-7 27002521 PMC5097109

[65] Grant BF, Stinson FS, Dawson DA, Chou SP, Dufour MC, Compton W, et al Prevalence and co-occurrence of substance use disorders and independent mood and anxiety disorders: results from the National Epidemiologic Survey on Alcohol and Related Conditions. Archives of general psychiatry. 2004;61(8):807–16. 10.1001/archpsyc.61.8.807 15289279

[66] Merikangas KR, McClair VL. Epidemiology of substance use disorders. Human genetics. 2012;131(6):779–89. 10.1007/s00439-012-1168-0 22543841 PMC4408274

[67] Davis L, Uezato A, Newell JM, Frazier E. Major depression and comorbid substance use disorders. Current opinion in psychiatry. 2008;21(1):14–8. 10.1097/YCO.0b013e3282f32408 18281835

[68] Brenner P, Brandt L, Li G, DiBernardo A, Boden R, Reutfors J. Treatment-resistant depression as risk factor for substance use disorders-a nation-wide register-based cohort study. Addiction (Abingdon, England). 2019. 10.1111/add.14596 PMC6593719 30938020

[69] Choi-Kain LW, Finch EF, Masland SR, Jenkins JA, Unruh BT. What Works in the Treatment of Borderline Personality Disorder. Current behavioral neuroscience reports. 2017;4(1):21–30. 10.1007/s40473-017-0103-z 28331780 PMC5340835

[70] Yu M, Linn KA, Shinohara RT, Oathes DJ, Cook PA, Duprat R, et al Childhood trauma history is linked to abnormal brain connectivity in major depression. Proc Natl Acad Sci U S A. 2019;116(17):8582–90. 10.1073/pnas.1900801116 30962366 PMC6486762

